# Reviews of Dental Literature

**Published:** 1904-07

**Authors:** 


### Reviews of Dental Literature.

Oxygenated Dentifrices. New Calcium and Oxygen Compound introduced into Dentistry.—At a meeting of the New York Odontological Society, held at the Academy of Medicine, 17 West Forty-third Street, New York, on Tuesday evening, April 19, Eustace H. Gane, of New York, the secretary of the Scientific Section of the American Pharmaceutical Association, gave a demonstration of the properties and dental applications of some new oxygen compounds.

Mr. Gane pointed out that considerable attention was being devoted, by chemists and physicians, to the problem of manufacturing substances which would readily part with oxygen, on account of the value of this element in its nascent state as a harmless germicide and antiseptic. The only compound hitherto available was hydrogen dioxide, which could be prepared only in the form of dilute solutions. Quite recently the ingenuity of American chemists had resulted in the discovery of several new compounds, which were available both for the dentist's and the physician's use. Thanks, too, to the development of the electric industries at Niagara, it was now possible to prepare these on a commercial scale at a reasonable cost. There were two of these oxygen compounds in particular which promised to be of great value to the dentist. These were the sodium and oxygen compound, which had been called "natrozone," and the calcium and oxygen compound, which was called "calox."

Mr. Gane showed a number of interesting experiments to illustrate the ease with which these compounds gave up oxygen. The most luminous one was the gentle heating in a glass tube of some iron filings with the calcium compound, when the oxygen united so rapidly with the iron as to raise the latter to a white heat.

The sodium and oxygen compound would bleach teeth discolored by age, decay, or even smoking, restoring them to their pristine whiteness without destroying the pulp, for, while oxygen was death to germs and destroyed dead organic matter, it was without action on healthy cells or tissues.

The calcium and oxygen compound would make an excellent tooth-powder, and, the speaker said, if the public could be induced to use such a preparation, which would not only clean the teeth, but sterilize the mouth, it was not too much to say that most of them would be saved from the terrors of the dental chair.

The paper was discussed by Dr. D. W. Ward, Professor of Chemistry at the New York Dental School, and by T. J. Keenan, associate editor of the *American Druggist.*

Mr. Keenan said that, in his long experience as a pharmacist, he had seen the rise and fall of many a much-vaunted dentifrice compound. Pharmacists had dentists to thank for many a new and wonderful mixture, each succeeding one of which was to be the acme of perfection, as regards antiseptic and detergent power. A simple compound of camphor and chalk, in the proportion of one part of the former to three of the latter, long held sway, but the use of some detergent, such as soap or alkali, later came to be considered indispensable, and some awful messes resulted in consequence. Charcoal was popular for a time on account of its supposed deodorant property, but it is not much used nowadays, as much because it caused discoloration by getting between the gums and the teeth as from a growing scepticism regarding its antiseptic value.

The speaker said that pharmacists were indebted to the dental profession for many useful suggestions regarding the nature of desirable additions to tooth-powders, as well as regarding what should be left out. For example, it was not so long since pharmacists had learned that the use of gritty and abrasive substances, like pumice-stone and cuttlefish-bone, was highly objectionable, because of the abrading action which such substances exerted on the delicate enamel of the teeth.

Of late years the main effort in the formulation of dentifrice recipes had been directed to the bringing together of ingredients which would, upon application to the teeth and gums, produce a refreshing sense of coolness, combined, of course, with real antiseptic power. The acid dentifrice of the French Codex was, he said, a good example of this class of compounds, consisting, as it did, of a mixture of cream of tartar and sugar of milk highly flavored with oil of peppermint. One ingenious investigator had hit upon potassium chlorate as a valuable addition to tooth-powders, being doubtless impelled to its use by a knowledge of its well-known antiseptic properties, and possibly having a vague idea that it would part with its oxygen readily. The antiseptic action of potassium chlorate, when applied as a tooth-powder, was more or less chimerical, and powders of this kind never became really popular, for what the laity wanted in a tooth-powder was a combination of substances and flavors that would leave a pungent and slightly sweetish taste in the mouth.

Referring to Mr. Gane's application of the medicinal peroxides as dentifrice compounds, Mr. Keenan said that this clearly marked a new era in the history of dentistry. The discovery was more important, as a matter of fact, than the discovery of radium, for it was more practical. When calcium dioxide, applied as a dentifrice to the teeth and gums, was brought into contact with the fluids of the mouth, it was split up into milk of lime and hydrogen dioxide, so that an antacid as well as a germicidal action was attained, the hydrogen dioxide being, of course, decomposed into water and liberating oxygen, which was supposed to combine with any organic matter that might be present in the teeth. He congratulated the New York Odontological Society in being selected as the medium through which the first announcement of the discovery was to be made public.—*American Druggist.*

The Mechanism of the Eruption of the Teeth.[1] By J. Thornton Carter, L.D.S. (Eng.), F.Z.S.

Whilst studying the teeth and jaws of fishes I was much struck by the fact that in all the skulls I had the opportunity of observing and examining, if the successional teeth were formed in crypts, a foramen always existed leading to the reserve tooth and transmitting a band of fibrous tissue to the capsule of the tooth. In tooth-bearing reptilia I have observed the same condition, and in mammalia it is a common feature. That these are not merely nutrient foramina seems evident, for the bundles of fibrous tissue which they transmit do not appear to be very vascular.

It would seem strange if a rudiment, absolutely functionless, were to be found so widely distributed throughout the animal kingdom, and I am of the opinion expressed by the "older anatomists" that the gubernaculum is concerned in some way in directing or effecting the eruption of the teeth.

The manner in which this takes place I shall endeavor to state briefly in the remainder of this paper, and though there may be auxiliary forces operating, such as blood-pressure, as Mr. Constant has so ingeniously brought forth, I believe the most important factor, and the only one which provides a satisfactory working hypothesis for the practitioner in dealing with cases of irregularity of the teeth, to be the force exercised on the tooth through the gubernaculum. This motive force I believe to be derived from absorption and deposition of bone (not merely of the free edges of the alveolus) operating on the muco-periosteum of the mouth, and to make clear how this takes place it is necessary for me to trespass on your time and patience by briefly outlining the mode of growth of the jaws.

Owing to the facilities afforded by the mandible for the investi-

[1] Abstract taken from the British Dental Journal of February, 1904.

gation of this subject, it has been mainly on this bone that the inquiry has been carried out, but the method of growth in the maxilla has been found to be of a similar nature.

The invaluable researches of the late Sir John Tomes, whom we revere as the founder of our profession as a scientific body, and whose work should never be out of reach of the earnest practitioner, show that the arch occupied by the deciduous teeth corresponds very closely with that occupied by their immediate successors, and that any alteration is referable "to deposition on the exterior surface and not to any fundamental alteration or interstitial growth."

Moreover, he found that the distance between the lingual surfaces of the second deciduous molars is almost the same as that between their successors, the second bicuspids or premolars; and drawing a line forward to the spina mentalis from the centre of a line connecting these latter teeth, he found the length to be about the same in the child where the deciduous molars were standing as in the case of the adult. But if the line were produced to the external alveolar plate a great difference would be apparent; "in point of fact, contemporaneously with the development of the crypts of the permanent teeth inside them, the temporary teeth and their outer alveolar plate are slowly pushed outward, a process the results of which we see in the separation which comes about between each one of the temporary teeth prior to their being shed, when the process of dentition is carried on in a perfectly normal manner."

His researches have shown that the growth of the mandible takes place mainly by deposition on the posterior border of the ascending ramus with coincident absorption over its anterior border, and by additions to the outer surface of the horizontal ramus.

Also, that the portion of the mandible lying below the level of the inferior dental canal practically reaches its full development, excepting the angle, by the age of seven years, but that the portion above the canal undergoes great change between this time and adult age.

During these years the vertical growth of the horizontal ramus takes place almost entirely in the alveolar portion,—*i.e.*, that portion which depends for its existence on the presence of teeth; for when these are lost this part of the bone is almost completely absorbed. There is also a considerable increase in the depth of the ascending ramus by deposition under the interarticular fibro-cartilage, which in this position partakes somewhat of the nature of an epiphyseal cartilage.

This constant and rapid growth of the anterior surface of the horizontal ramus and more rapid but intermittent growth of the alveolar portion of the bone causes the muco-periosteum covering the jaw to be at times in a state of considerable tension. That this is so is apparent on looking into the mouth of a child between the years of two and seven, and that this tension exercises a force leading to change of position of teeth is plainly seen. To take a single example in the case of the first permanent molar: at the age of two years the follicles of the deciduous molars lie immediately over the tooth-sacs of the first permanent molars. As the jaw lengthens backward these tooth-sacs are borne backward by the tension and consequent extension of the muco-periosteum to which they are attached.

As before mentioned, the deciduous teeth are never completely roofed in by their crypts, but lie in incomplete alveoli; as the growth of the jaw goes on, the tension on the anterior fibres causes absorption to start at the anterior border of the crypt, this being the part where pressure first pulls. This absorption continues until the tension is relaxed and the tooth free to emerge from its crypt, when it is gradually raised to the surface by the constant thickening of the outer alveolar plate operating on the membrane covering the jaw, the posterior alveolar plate acting as a more or less fixed *point d'appui.*

The edges of the crypt, relieved from the pressure which caused their absorption, immediately assume a period of great activity (contrary to the steady continuous growth of the outer plate), growing up around the neck of the tooth to afford it support, prevent its displacement, and to protect the remainder of the tooth-germ during its completion.

Meanwhile, the calcification of the permanent teeth and the steady growth of the jaw continue, until the mucous membrane of the mouth is again in a condition of tension. This causes the peridental membrane ensheathing the root of the deciduous tooth to change its function, and, since steady pressure causes absorption, either to become or to give origin to an absorbent organ, the absorption starting at the point where the pressure is greatest.

Coincidentally with the process of absorption there is a deposition of bone immediately beneath, so that ultimately the crown alone remains attached to the gum and separated from the bone roofing over the crypt of its successor by the absorbent organ only.

As before stated, the follicle of the permanent tooth retains its connection with the mucous membrane of the mouth by means of the gubernaculum, the fibres of which pass upward through the iter dentis, and diverging radially blend with the fibrous elements of the gum.

The tension produced by the unequal growth of the jaw acts first on the anterior fibres of the gubernaculum, causing the labial margin of the iter dentis to be absorbed, and the crypt is open sufficiently to permit the egress of the partially formed tooth which is gradually raised to its place in the dental arch in the same manner as were the deciduous teeth.

We have another instance of a somewhat similar process taking place. In Heisler's "Text-book of Embryology" it is stated that in the case of the descent of the testis, the inguinal ligament or gubernaculum testis does not grow, whilst the surrounding tissues do grow, and that by this means it is displaced downward from its original position, attributing little or no part to the unstriped muscle fibres which it contains.

Comparative anatomy appears to lend support to the views enunciated above. In plagiostomous fishes (standing as they do at the very base of the vertebrate phyllum) the simplest conditions are found, and since the parts have not undergone great specialization, investigation of the process of development and eruption in this sub-order is comparatively simple when contrasted with the conditions obtaining in the more specialized groups, where a more highly specialized organ is needed leading to a corresponding complexity of the formative process.

The Uses of Swiss Broaches. By Otto E. Inglis, D.D.S., Philadelphia, Pa.

The Swiss broach is an invention from the watchmaker's art which has become almost a necessity in root-canal treatment. These broaches, as sold by the jeweller's finders, consist of a handle, or thumb-shank, like that on the ordinary barbel broach, and a foil-like polygonal blade, tapering to a fine point.

The broaches vary in size, from extremely fine to comparatively thick. They may be purchased of very hard temper and of very soft temper. The latter renders the broach useless except for barbing, and the former makes it so exceedingly brittle as to render it useless as purchased. The high temper may be very readily reduced, however, by placing a few broaches in a test-tube, and holding the latter over a flame, allowing the flame to impinge upon that part of the tube over which the shanks of the broaches lie. As the correct heat is approached, the blade of the instrument will change to a straw color, then to a bright blue. This change will appear first near the shank, and, if the tube be moved slowly, the color will creep along the blade until the extreme tip is tempered. The broach may then be thrown out to cool on anything that will not burn. The operation may be done with a single broach over a tiny flame, or near the flame of a match, but the results are much inferior, as a rule.

This tempering changes the broach from a useless article to one which may be tied in a knot without breaking, but may be used to penetrate a canal, offering some resistance, without the maddening result of bending upon itself into a crumpled mass of useless steel.

In the selection of the broaches, only those having a distinct taper to the blade should be bought, as those with blades of even diameter throughout are very liable to bend.

The uses of these little instruments are varied.

As explorers for fine points of exposure, they are admirable, entering the small orifice readily, and, while producing the sensation desired as a diagnostic, they do not make painful compression of the pulp.

As explorers of canal apertures for the determination of the size and direction of the canal, they are very useful, and may be filed to an extreme thinness to enable them to enter the finer canals.

As reamers of canals, they also do admirable work. Successive sizes, beginning with the finest, are passed into the canal and rotated with the fingers. Acids tend to injure these instruments, so that they may break, but this does not occur, as a rule, unless undue force be used.

As carriers of cotton for the swabbing out of canals for any object, they are indispensable, though of course an analogous instrument may be constructed by filing the fine truncated end of a Donaldson bristle to a polygonal form. Their value, in this connection, lies in their smooth taper and unround form, which permits the cotton to be quickly rolled upon them, and, when charged with the canal contents, to be removed; the cotton is readily removed without soiling the fingers by grasping the broach with the finger and thumb of the left hand at a point near the shank, and back of the loose fibres of cotton; the shank is held with the thumb and finger of the right hand, and with a quick pull the cotton is slipped off the broach as a neat cone.

The same instrument may be used to place cottons in canals as temporary dressings. This may be done by using the broach, mounted in a chuck handle, as a canal plugger, but the dressing is more easily done by another method, especially in the finer canals.

The cotton is wound on the broach, as previously described, with as few fibres about its end as possible, as all bulk interferes with canal penetration. If the canal be very fine, the broach which explored it may be filed down, and again smoothed by drawing it through a folded cuttle-fish disk. In dressing the canal, the broach, with the cotton upon it, is passed in with a twist to the right to engage the fibres with the canal walls; when solidly placed, a couple of twists to the left are made to loosen the broach from the centre of the cone. It is then withdrawn about a sixteenth of an inch, and then pressed in again, an act which crimps the cotton cone upon itself.

This may be done, even if the broach be bent so as to enter mesial canals of lower molars. If desired, the broaches may be many times bent and again straightened without injury, but it is well to avoid too much of this without annealing, as all steel is finally injured by the disarrangement of its molecules.

The rolling of the cotton upon the broach is done by first laying a few loose fibres upon the forefinger of the left hand. The broach is placed upon it, allowing a few fibres to lie beyond its end. Next, the thumb is lightly pressed upon the broach. The shank is then quickly rotated with the right thumb and forefinger, and, as the cotton is rolled up, the left thumb and forefinger are used to stroke it into a symmetrical cone. If the pointed broach be used, it will be noted that there is a tendency on its part to penetrate the end of the cone of cotton. This may be obviated by slightly truncating the end of the broach with scissors. This penetration of the cotton is annoying from the fact that apical tissues are liable to be irritated, and that the broach is liable to be stripped of the cotton which crowds back upon it as it comes into friction with the canal walls. In use as a swab-carrier, the broach is constantly twisted to the right, if twisted at all. To remove cotton, it may be twisted in this manner into the loose fibres until these are tightly engaged; but, as a rule, an old, barbed broach, or Donaldson cleanser, is preferable for this purpose.

Any one who has used as a cotton-carrier a broach which requires a wire brush, or burning off, to free it, will be much relieved by the adoption of this method.—*Stomatologist* for November.

